# Model-based optimization of G-CSF treatment during cytotoxic chemotherapy

**DOI:** 10.1007/s00432-017-2540-1

**Published:** 2017-11-04

**Authors:** Sibylle Schirm, Christoph Engel, Sibylle Loibl, Markus Loeffler, Markus Scholz

**Affiliations:** 1 Medical Faculty, Institute for Medical Informatics, Statistics and Epidemiology (IMISE), University of Leipzig, Haertelstraße 16-18, 04107 Leipzig, Germany; 2 German Breast Group, c/o GBG Forschungs GmbH, Martin-Behaim-Straße 12, 63263 Neu-Isenburg, Germany

**Keywords:** Cytotoxic drugs, Filgrastim, Pegfilgrastim, Leukopenia, Neutropenia, Risk-adapted treatment

## Abstract

**Purpose:**

Although G-CSF is widely used to prevent or ameliorate leukopenia during cytotoxic chemotherapies, its optimal use is still under debate and depends on many therapy parameters such as dosing and timing of cytotoxic drugs and G-CSF, G-CSF pharmaceuticals used and individual risk factors of patients.

**Methods:**

We integrate available biological knowledge and clinical data regarding cell kinetics of bone marrow granulopoiesis, the cytotoxic effects of chemotherapy and pharmacokinetics and pharmacodynamics of G-CSF applications (filgrastim or pegfilgrastim) into a comprehensive model. The model explains leukocyte time courses of more than 70 therapy scenarios comprising 10 different cytotoxic drugs. It is applied to develop optimized G-CSF schedules for a variety of clinical scenarios.

**Results:**

Clinical trial results showed validity of model predictions regarding alternative G-CSF schedules. We propose modifications of G-CSF treatment for the chemotherapies ‘BEACOPP escalated’ (Hodgkin’s disease), ‘ETC’ (breast cancer), and risk-adapted schedules for ‘CHOP-14’ (aggressive non-Hodgkin’s lymphoma in elderly patients).

**Conclusions:**

We conclude that we established a model of human granulopoiesis under chemotherapy which allows predictions of yet untested G-CSF schedules, comparisons between them, and optimization of filgrastim and pegfilgrastim treatment. As a general rule of thumb, G-CSF treatment should not be started too early and patients could profit from filgrastim treatment continued until the end of the chemotherapy cycle.

**Electronic supplementary material:**

The online version of this article (10.1007/s00432-017-2540-1) contains supplementary material, which is available to authorized users.

## Background

The haematopoietic growth factor G-CSF is routinely used in cancer therapy to prevent or ameliorate leukopenic conditions. Its effectiveness has been shown in several studies (Kosaka et al. 2015; Lee et al. 2013; Vogel et al. 2005; Altwairgi et al. 2013; Dale 2002, 2003; Kuderer et al. 2007; Crawford et al. 1991; Bohlius et al. 2008; Sung et al. 2007; Cooper et al. 2011; Mhaskar et al. 2014; Clark et al. 2003, 2005). Although G-CSF is expensive, its application often results in an overall cost-reduction due to the reduced number of severe events (Zagonel et al. 1994; Wang et al. 2016).

With the introduction of G-CSF support, more intense chemotherapies became feasible in order to improve outcome of patients (Trumper et al. 2008; Untch et al. 2011a, b; Pettengell et al. 1992; Pfreundschuh et al. 2004a, b, 2008; Diehl et al. 2003; Sieber et al. 2003; Blayney et al. 2003, 2005). A number of G-CSF pharmaceuticals are in use differing in both, pharmacokinetic and pharmacodynamic properties (Kuwabara et al. 1994, 1996a, b; Yang et al. 2004; Zamboni 2003; Molineux 2002; Houston et al. 1999). Various generics are available or under development.

Several in vivo modes of action of G-CSF are known, namely increased proliferation, accelerated maturation and improved release of mature bone marrow granulopoietic cells (Lord et al. 1989; Schmitz et al. 1993). In combination with the relatively short half-life of blood granulocytes and the bone-marrow suppressive effects of cytotoxic chemotherapy, application of G-CSF results in complex dynamics of blood granulocytes which cannot easily be predicted. As a consequence, optimal G-CSF support for a given chemotherapy and patient population is a non-trivial task. It depends on a large number of variable therapy parameters such as the type of cytotoxic drugs, granulotoxic risk factors of patients, type of G-CSF derivative applied and its dosing and timing (Bennett et al. 2013).

In clinical trials, it is practically impossible to control for each of these factors. Therefore, only limited attempts were made to compare the efficacy of different G-CSF schedules in the context of clinical trials (Danova et al. 2009; Holmes et al. 2002; Loibl et al. 2011; Lyman et al. 2009; Vose et al. 2003; Zwick et al. 2011; Faber et al. 2006; Crawford et al. 1997; Leonard et al. 2015). However, available clinical trials showed that considerable improvements can be expected by optimized G-CSF schedules. Since such trials are both, cost and time-intensive, there is relevant need to predict the outcome of alternative G-CSF schedules prior to clinical application. On the basis of large clinical and experimental data sets, we developed a comprehensive biomathematical model of human granulopoiesis including detailed information on injection, pharmacokinetics and pharmacodynamics of both, chemotherapeutic drugs and three G-CSF derivatives namely filgrastim, pegfilgrastim and the experimental drug Maxy-G34 (Scholz et al. 2005, 2009a, 2009b, 2012; Chua et al. 2014; Engel et al. 2004; Schirm et al. 2013, 2014b). The model was validated in several settings and is now ready to make clinically relevant predictions regarding G-CSF schedules optimized for given chemotherapeutic regimens.

In this paper, we present our approach for developing optimized dosing and timing schedules of G-CSF for a variety of applications, i.e. for different chemotherapy schedules, risk groups of patients and usage of filgrastim or pegfilgrastim. Different measures of treatment outcome are considered. We also show examples of model predictions validated in the context of clinical trials.

## Methods

### Model of human granulopoiesis

**Fig. 1:**
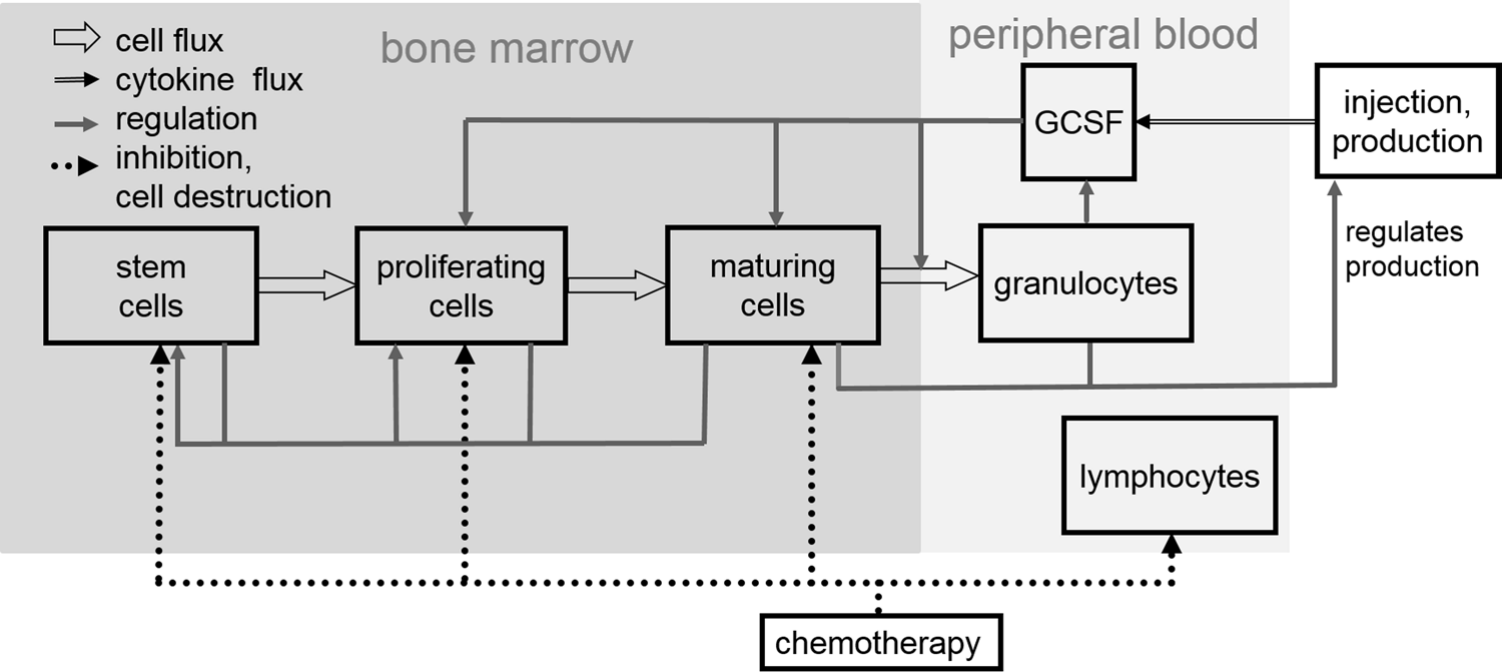
Schematic representation of human granulopoiesis model under chemotherapy and G-CSF treatment. Boxes represent major cell- or cytokine compartments of the model. We modelled two G-CSF derivatives, filgrastim and pegfilgrastim. Arrows represent cell/cytokine fluxes and interactions

We first introduce our biomathematical model of human granulopoiesis under chemotherapy and G-CSF support which is used to optimize G-CSF treatment during cytotoxic chemotherapy later. The model consists of a set of coupled differential equations describing time dependence of major bone marrow cell stages, circulating cells, cytokines at various sites, corresponding stimulation of bone marrow and toxic effects of chemotherapy. Treatments with G-CSF (filgrastim, pegfilgrastim) and chemotherapy (10 different chemotherapies, 33 different schedules for treatments for a variety of cancers) are modelled (Schirm et al. 2014b). Without any therapeutic intervention, a stable steady-state of all cell and cytokine compartments is achieved. The general structure of the model is shown in Fig. 1.

Equations can be attributed to three major mechanisms namely cell kinetics of bone marrow granulopoiesis, pharmacokinetics and pharmacodynamics of endogenous G-CSF and G-CSF pharmaceuticals (filgrastim, pegfilgrastim) and chemotherapy action. We briefly describe these major parts of the model and corresponding biological assumptions in the following.

The cell kinetic granulopoiesis model is explained in detail in Scholz et al. (2012). Here, we briefly sketch the main assumptions:

1. Granulopoietic cells originate from a pluripotent stem cell compartment. Cell division can create new stem cells or cells committed to granulopoietic lineage. All other haematopoietic lineages are neglected.
2. Subsequent proliferation and maturation of granulopoietic cells are described by transitions of cells from the stem cell compartment to the proliferating compartments of progenitor cells, proliferating precursors (PGB), and finally, maturing precursor cells which are unable to proliferate (MGB). For the latter, a postmitotic amplification is assumed which describes a loss of mature granulocytes in bone marrow due to apoptosis (Mackey et al. 2003). Finally, granulocytes are released to circulation (GRA). The system is regulated by several feed-back loops of which G-CSF is the strongest mediator.
3. The changes of the compartment sizes ${\frac{{{\text{d}}C(t)}}{{{\text{d}}t}}}$ in compartments *X* are determined by balance equations of cell influx rate $C_{X}^{\text{in}} (t)$ from the preceding compartment, cell amplification $A(t)$ (if applicable), cell efflux dependent on the transition time $T(t)$, and loss rate ${\varPsi_{\text{total}}^{X} }(t)$ caused by cytotoxic chemotherapy and named toxicity function in the following (Schirm et al. 2014b):

$${\frac{{{\text{d}}C_{X} (t)}}{{{\text{d}}t}} = C_{X}^{\text{in}} (t) \times A(t) - \frac{{C_{X} (t)}}{T(t)} - \varPsi_{\text{total}}^{X} (t)C_{X} (t)}.$$

Increased G-CSF serum concentration results in higher amplification rate and longer transition time in the compartment PGB, and reduced transition time and apoptosis rate in MGB.

### Pharmacokinetic model of G-CSF applications

The most frequently used derivatives filgrastim and pegfilgrastim differ substantially in their pharmacokinetic properties. This is addressed by a general pharmacokinetic model of G-CSF injections developed for humans recently (Scholz et al. 2012):

1. Three compartments are modelled: G-CSF is injected into the subcutaneous compartment. In the central compartment, G-CSF is haematologically active. The peripheral compartment represents reversible binding of G-CSF (Scholz et al. 2005, 2012).
2. The influx of G-CSF from the subcutaneous compartment into the central compartment is delayed (Kota et al. 2007). This is modelled by splitting the subcutaneous compartment into two subcompartments.
3. Reversible bindings of G-CSF are modelled by transitions between central and peripheral compartment using first-order kinetics (Kuwabara et al. 1996b).
4. Endogenous production of G-CSF is regulated by the demand of mature granulocytes (Scholz et al. 2005).
5. Bioavailability of G-CSF is assumed to be dose-dependent. Thus, a part of the applied G-CSF is removed from the injection compartment by a Michaelis–Menten kinetic.
6. From the central compartment, G-CSF is irreversibly removed by two processes: a first-order kinetic describing the unspecific renal elimination (Kuwabara et al. 1996b) and a Michaelis–Menten kinetic describing the specific elimination by circulating granulocytes.
7. Differences in G-CSF derivatives filgrastim and pegfilgrastim are modelled by different settings of pharmacokinetic and pharmacodynamics parameters, i.e. parameters of the G-CSF-mediated regulatory feed loops (Harris and Chess 2003; Sarkar et al. 2003; Veronese and Mero 2008; Scholz et al. 2009b). Parameter values for filgrastim and endogenous G-CSF are the same.

### Chemotherapy model

The impact of cytotoxic chemotherapy on haematopoiesis is modelled by drug, drug-dose and cell-stage specific toxicity functions in the following way (Schirm et al. 2014b):

1. A set of concatenated first-order transitions is used to model a delayed maximum of cell damage after the injection of chemotherapeutic drugs (Schirm et al. 2013).
2. When chemotherapeutic drugs were applied for the first time, we assume a somewhat higher toxicity than for further injections.
3. If multiple drugs are applied simultaneously, we add the corresponding toxicity functions to calculate the overall toxic effect.
4. Depletion of lymphocytes (LY) is phenomenologically modelled by an additional toxicity equation with two parameters.
5. The chemotherapy effect is assumed to be reversible. All cell-kinetic parameters remain unchanged.
6. Often, prednisone is applied to avoid tumour lysis syndrome. Prednisone is assumed to cause a prolonged half-life of granulocytes, and therefore, it temporarily increases granulocyte counts.

### Data and model calibration

The model was parametrized on the basis of clinical data of 10 different chemotherapies. These chemotherapies are used to treat patients with NHL (non-Hodgkin’s disease), HD (Hodgkin’s lymphoma), BRCA (breast cancer), NSCLC (non-small cell lung cancer), and DLBCL (diffuse large B cell lymphoma). An overview is presented in Table S1 in Additional file 1. Data were retrieved from published figures using the software tool “YCASD” (Gross et al. 2014) or directly from the clinical trial databases of cooperating clinical study groups. Model equations contain parameters for which often no direct biological data are available. This especially applies for parameters quantifying chemotherapy toxicity on bone marrow. These parameters were determined by fitting the model to the above-mentioned data sets as described elsewhere (Rechenberg 1973, 1994). Model parameters were validated on the basis of data sets not used for parameter fitting (Schirm et al. 2014b; Scholz et al. 2012).

### Application to risk groups

Several risk factors associated with the degree of leukopenia under chemotherapy have been identified and validated in different settings. This comprises for example pre-therapeutic risk factors such as age, sex, WHO performance status as well as intra-therapeutic risk factors such as toxic response in the first therapy cycle. A risk model accounting for these factors was proposed by Ziepert et al. (2008) for CHOP chemotherapy. The risk model is implemented in a web-based tool (see http://www.toxcalculator.com). We used the tertiles of the risk score to divide our study population into low, medium and high-risk group and propose different G-CSF schedules for them in the following.

In order to apply our model to different risk groups, we assume that parameters regarding pharmacokinetics and pharmacodynamics of G-CSF as well as cell-kinetic parameters are constant among risk groups, but the parameters regarding chemotherapy toxicity might differ. This is motivated by observed heterogeneities regarding metabolism of cytotoxic drugs (Bruno et al. 1997; Kloft et al. 2006; Bennett et al. 1987; Sulkes and Collins 1987; Iyer and Ratain 1999; Rushing et al. 1994).

Applying this paradigm, we can derive risk-specific toxicity parameters of patients by fitting the predictions of the model to available data of the subgroups (Table S2 in Additional file 1).

### Model simulations of new chemotherapy schedules

A key feature of the model is that new, yet untested schedules of both, chemotherapy and G-CSF treatment can be simulated. This requires that toxicity parameters of the chemotherapy are available for the population of interest, i.e. data of patients under at least one G-CSF schedule (or no G-CSF) of the chemotherapy of interest are required allowing to estimate the toxicity parameters thereon. Then, alternative timing of chemotherapy or G-CSF, alternative derivatives and dosing of G-CSF can be simulated by the model. An overview of already available toxicity parameters is listed in an earlier publication of our group (Schirm et al. 2014b).

### Optimization of therapy schedules

**Fig. 2:**
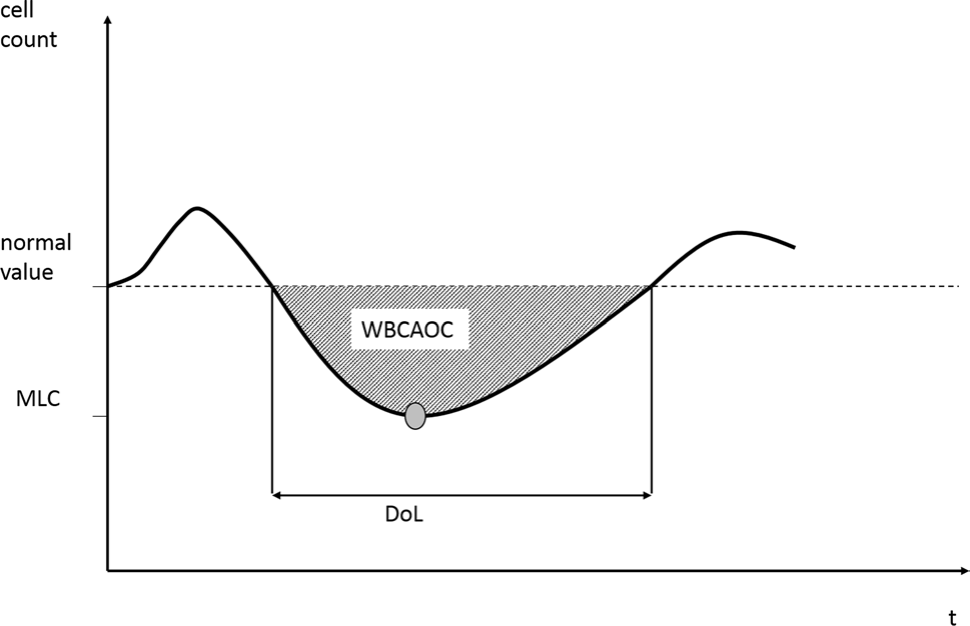
Illustration of toxicity outcomes. We consider the area over the curve (WBCAOC), minimal cell count (MLC) and duration of low cell counts (DoL) as measures of severity of leukopenia. We treat 4.000 leukocytes/µL as normal value for this purpose

To compare the performance of alternative G-CSF schedules for a given chemotherapy schedule of a given population of patients, we calculate a number of quantities mirroring the overall leukotoxicity: The area over the curve of white blood cells (WBCAOC) measures the area between simulated cell counts and the value of 4000 leukocytes/µL. There is some evidence that the risk of infectious complications in patients depends on the depth and the duration of leukopenia (Crawford et al. 2004; Li et al. 2016). We separately consider the duration of leukopenia (DoL) referring to the cumulative time of leukopenia and the minimal leukocyte count (MLC) referring to the minimal cell count throughout all chemotherapy cycles as alternative endpoints of G-CSF optimization (Fig. 2).

### Technical implementation

The model equations were programmed and solved on a standard personal computer using the numeric computation software Matlab 7.5.0.342 and the integrated Simulink toolbox v7.0 (The MathWorks, Natick, MA). Model simulations were performed by numerical integration of the ODE system using the variable step solver from Adams and Bashford (ode113).

## Results

### Validated model predictions

**Fig. 3:**
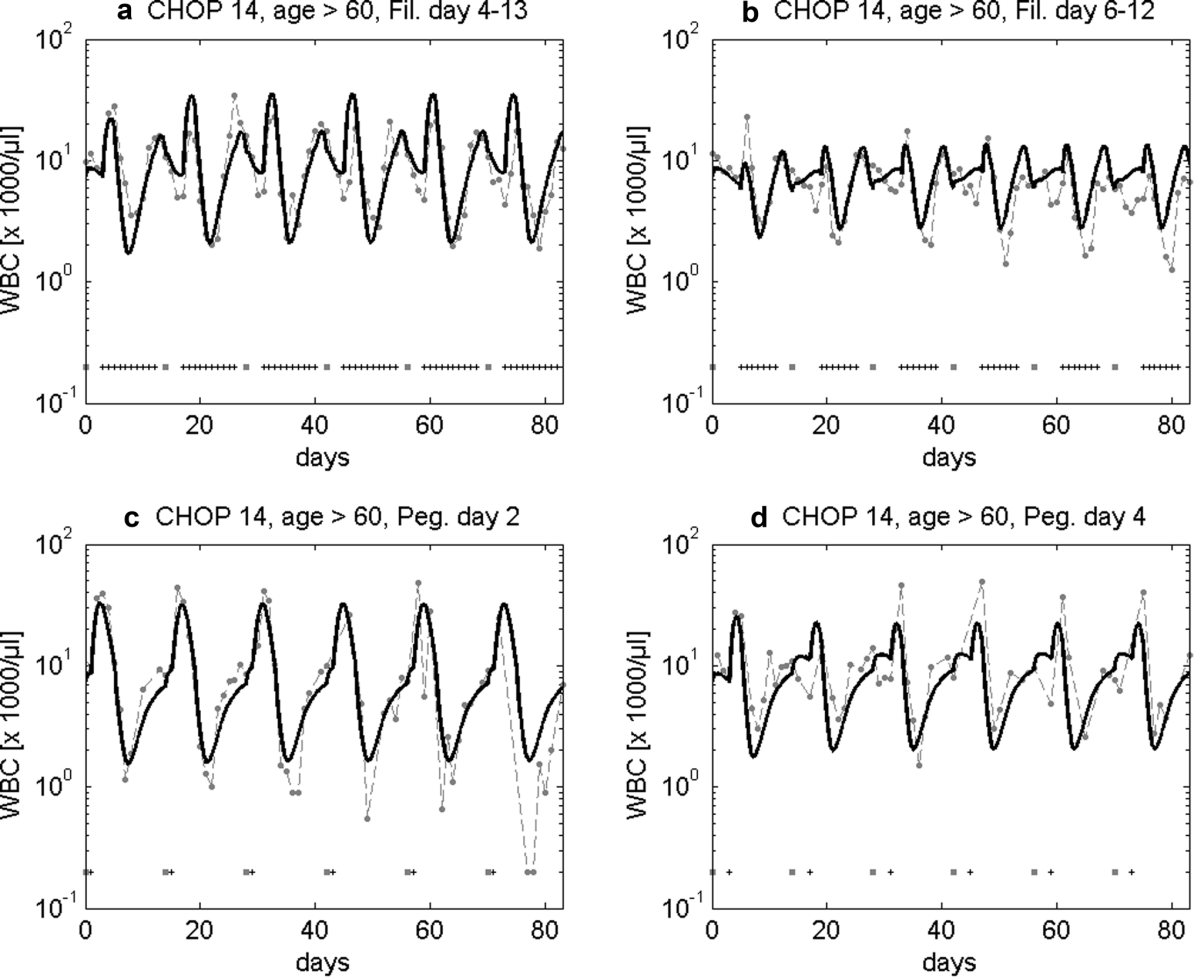
Validation of model predictions. We compare model and data for six cycles of CHOP-14 for elderly patients either treated with filgrastim at cycle days 4–13 (**a**) or 6–12 (**b**). Dots represent patient medians, squares correspond to chemotherapy administrations, “+” correspond to days with G-CSF injections. Model predictions fit well to data of the RICOVER-60 trial and the NHL-B trial and show that the reduced G-CSF schedule is feasible (Schirm et al. 2014b; Zeynalova et al. 2013; Pfreundschuh et al. 2004a, 2008). We further predicted that later pegfilgrastim application is advantageous for CHOP-14 chemotherapy of elderly NHL patients. This was compared in a randomized trial of pegfilgrastim day 2 (**c**) vs. day 4 (**d**) (Zwick et al. 2011). Again, a good agreement of model and data was observed (Schirm et al. 2014b)

In the past, we showed that our model can successfully predict the granulotoxic outcome of CHOP chemotherapy with G-CSF support. Some of these predictions were already tested in clinical trials resulting in improved filgrastim and pegfilgrastim schedules for CHOP chemotherapy. We present these successful predictions in brief. For CHOP-14 therapy in elderly patients we predicted that a reduced filgrastim schedule with applications at days (d) 6–12 of each cycle is also feasible compared to the standard d4–13 protocol. This was confirmed by retrospective analyses of the RICOVER trial in which both schedules were applied. We present the comparison of model and data for these scenarios in Fig. 3a, b. Interestingly, a recent simulation of Craig et al. (2015) also predicted that later start of filgrastim treatment could be advantageous for general 14 day schedules and that fewer injections are required in this case.

We also predicted that later applications of pegfilgrastim are advantageous compared to early applications in CHOP-14 regimen of elderly NHL patients. This was confirmed in the pegfilgrastim trial comparing pegfilgrastim applied at d2 with d4 which resulted in a clear advantage of the latter with respect to leukocyte nadir, days with leukocytes < 2 × 10^3^/mm^3^, grade 3 and 4 leukocytopenias, grade 4-only leukocytopenias, grade 3 and 4 infections, deaths during leukocytopenia and interventional antibiotics (Zwick et al. 2011). It turned out that our predictions were not only qualitatively correct, but also in good quantitative agreement with the observed clinical data (Fig. 3c, d).

### Optimization of chemotherapy schedules without considering risk factors

We now use our model to make predictions regarding optimal filgrastim schedules of a number of established and novel chemotherapy schedules. An overview of all optimized G-CSF schedules and corresponding WBCAOC values is presented in Table 1. Results for the other endpoints, DoL and MLC, can be found in Table S3 in Additional file 1.

**Table 1:** Predicted WBCAOC values of different simulated G-CSF schedules, chemotherapies and risk groups

Therapy	Risk group	Optimal start of Fil	Optimal #Injection Fil	Optimal outcome value Fil	Optimal start of Peg	Optimal outcome value Peg	Currently used schedules	Current outcome value
CHOP-14 elderly	High	7	8	50.51	7	57.16	Fil d4–13	72.58
							Fil d6–12	77.92
							Peg d2	112.45
							Peg d4	91.10
	Medium	9	6	2.16	7	2.81	Fil d4–13	10.47
							Fil d6–12	7.55
							Peg d2	38.82
							Peg d4	22.14
	Low	8	4	0.00	7	0.00	Fil d4–13	0.00
							Fil d6–12	0.00
							Peg d2	21.06
							Peg d4	6.94
BEACOPP esc	All	7	15	62.07	7	52.98	Fil d8–15	145.16
ETC	All	7	8	0.70	6	5.34	Fil d3–10	14.86
CHOP-12 elderly	All	7	6	19.12	6	20.50	–	–

#### BEACOPP escalated

**Fig. 4:**
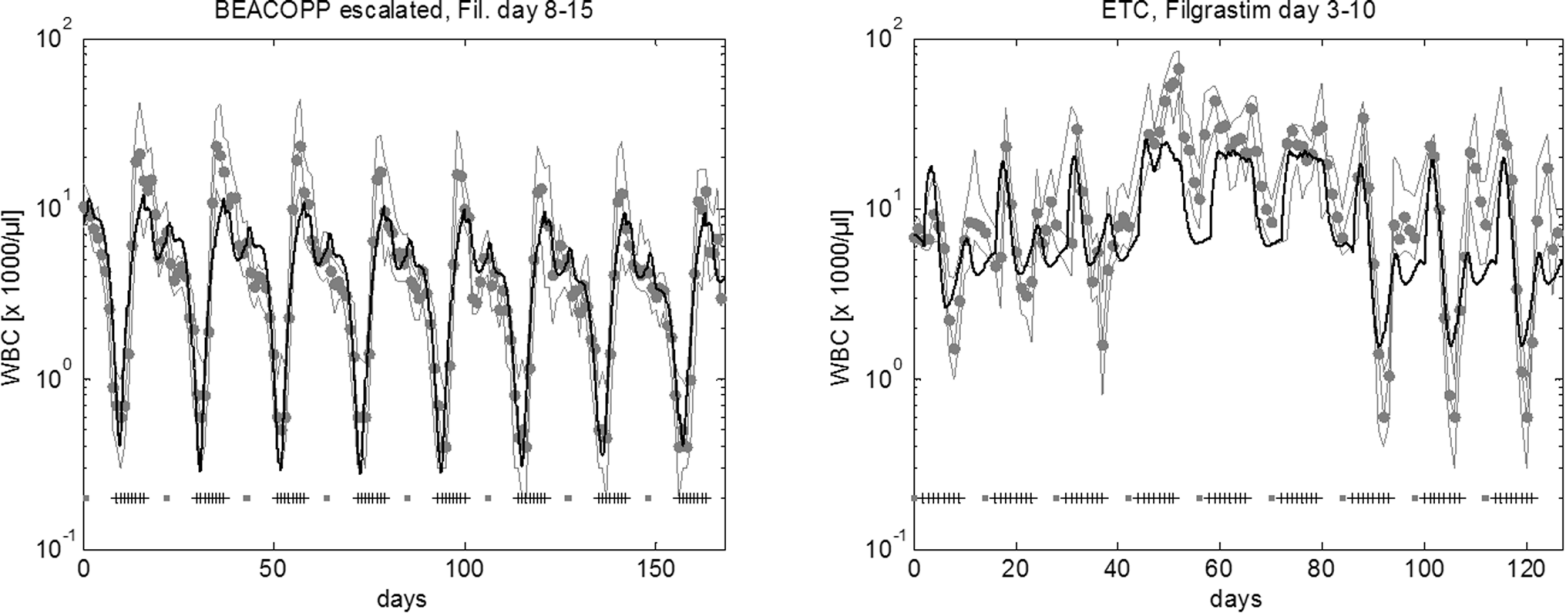
Agreement of model and data for BEACOPP escalated and for ETC. We consider eight cycles of BEACOPP escalated chemotherapy of Hodgkin’s lymphoma with filgrastim at cycle days 8–15 (left), and nine cycles of ETC adjuvant breast cancer chemotherapy with filgrastim at cycle days 3–10 (right). Dots represent patient medians, grey lines represent interquartile range of patient data, squares correspond to chemotherapy administrations, “+” corresponds to days with G-CSF injections

**Fig. 5:**
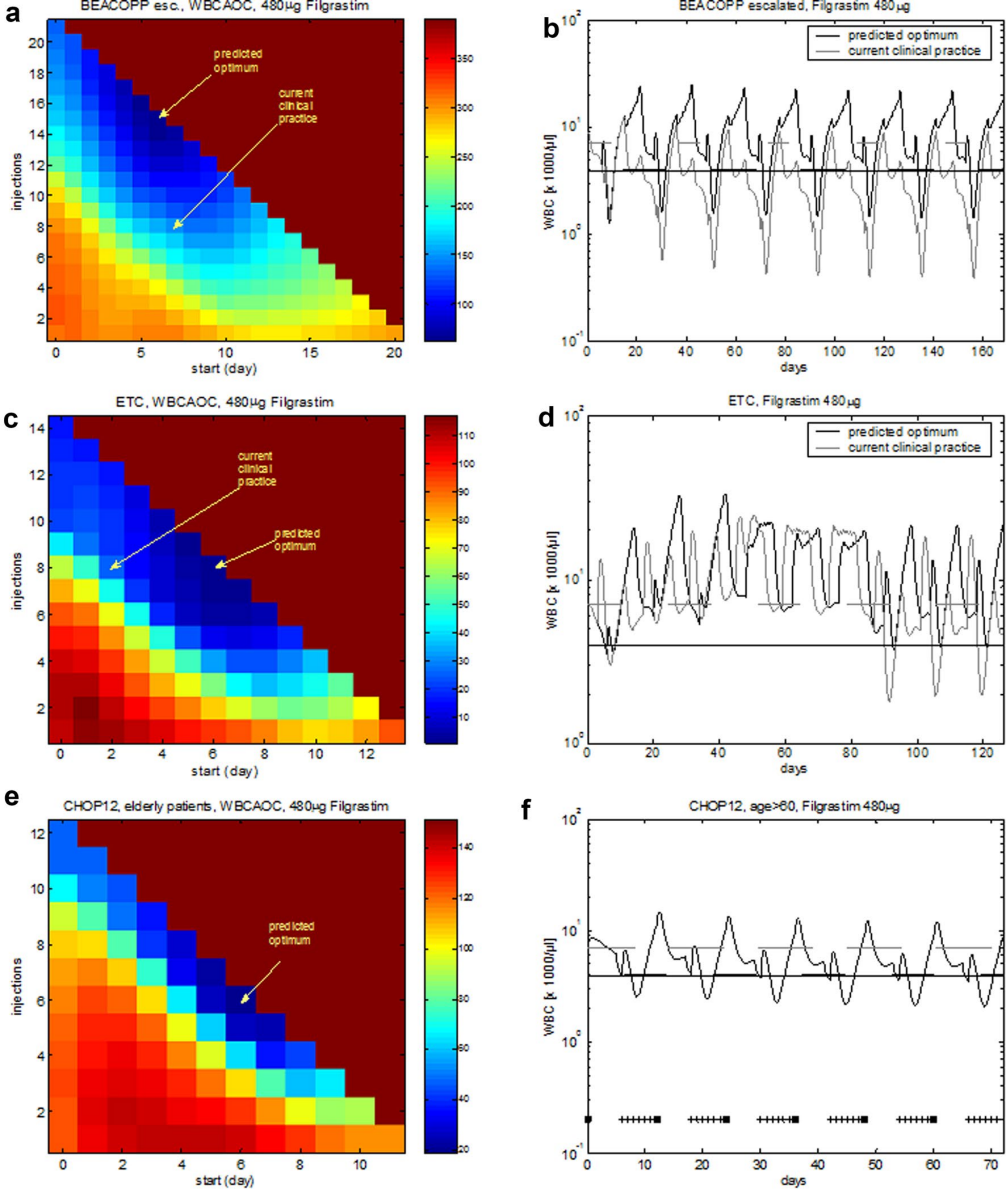
Optimized G-CSF schedules for a variety of chemotherapies. **a** Optimization of eight cycles of BEACOPP escalated with filgrastim. **b** We consider eight cycles of BEACOPP escalated with filgrastim at cycle days 8–15 (“current clinical practice”) and filgrastim at cycle days 7–21 (“predicted optimum”). **c**, **d** We consider nine cycles of ETC with filgrastim. Current clinical practice: filgrastim at cycle days 3–10. Optimization with the same G-CSF schedule in all cycles results in a predicted optimal G-CSF treatment at days 7–14. **e**, **f** CHOP-12 (hypothetical chemotherapy of elderly patients): predicted optimal filgrastim treatment is d7–12. **a**, **c**, **e** The *X*-axis denotes the starting day of filgrastim. The *Y*-axis shows the number of filgrastim injections. The colour corresponds to the calculated WBCAOC (blue: lower WBCAOC, red: high WBCAOC). Background colour: WBCAOC obtained without G-CSF treatment

Eight cycles of BEACOPP escalated is the German standard chemotherapy to treat intermediate and advanced stages of Hodgkin’s disease in younger patients (< 60 years). According to study protocols, it is recommended to apply filgrastim at d8–15 at each cycle. Figure 4 shows the predicted median leukocyte time course of this schedule. We now varied both, starting day of G-CSF treatment and number of injections in order to predict WBCAOC of these alternative schedules (Fig. 5a, b). It revealed that leukopenia prophylaxis could be improved by starting earlier (~ d6–7) and providing a higher number of injections (~ until the end of each cycle). We also considered substituting filgrastim by pegfilgrastim. We predict that in this case, pegfilgrastim should be applied at d6–7 to achieve optimal leukopenia prophylaxis.

#### ETC

The ETC regimen is used as adjuvant chemotherapy of breast cancer patients in the German Breast Group (Moebus et al. 2010). It consists of three consecutive cycles of epirubicin (E), paclitaxel (T), and finally, cyclophosphamide (C). Filgrastim was recommended to be applied at d3–10 of each cycle (Fig. 4). We predict that leukopenia prophylaxis can be clearly improved if G-CSF is applied at d6–13 (Fig. 5c, d, Table 1, Table S3). If pegfilgrastim is used instead of filgrastim, it should be applied at d6 of each cycle (Fig. S1).

However, since haematotoxic risk differs considerably between the chemotherapeutic drugs (lowest for T, highest for C), it appears to be worthwhile to modify G-CSF schedules according to the drug currently applied. We implemented a stepwise optimization for this sequential chemotherapy taking the different cytotoxicities of the drugs into account: in cycles 1–3, G-CSF should be applied from d6–10. In cycles 4–9, optimal G-CSF therapy starts on d7 with 8 injections (Fig. S1 in Additional file 1). The optimal WBCAOC is 0.57, while that of the current standard therapy is 14.9. The optimization assuming the same G-CSF schedule in each cycle (d7–14) yields 0.70. Thus, the improvement by cycle-specific G-CSF schedules is only moderate compared to the optimal unique G-CSF schedule, which probably does not outweigh the higher organizational effort.

#### CHOP-12

Densification of CHOP chemotherapy from cycle duration 21 to cycle duration 14 resulted in improved outcomes of elderly NHL patients (Pfreundschuh et al. 2004a). This densification was only possible by intense leukopenia prophylaxis with either filgrastim or pegfilgrastim. By model simulation, we analysed whether a further time intensification (CHOP-12) is feasible with respect to leukopenic risk and predict corresponding optimal G-CSF treatment.

We predict that CHOP-12 is best accompanied by G-CSF at d7-12 after chemotherapy start and that this results in tolerable toxicity. However, mild cumulative toxicity at later therapy cycles is observed (Fig. 5e, f).

### Optimization of G-CSF schedules considering individual risk factors

G-CSF schedules proposed for the previous chemotherapies were optimized for medians of patients, i.e. patient’s heterogeneity in toxic response was ignored so far. Here, we provide predictions for risk-adapted therapies using a recently proposed statistical model of leukopenia risk of elderly patients under CHOP treatment. Patients were divided into three risk groups according to tertiles of the risk score.

**Fig. 6:**
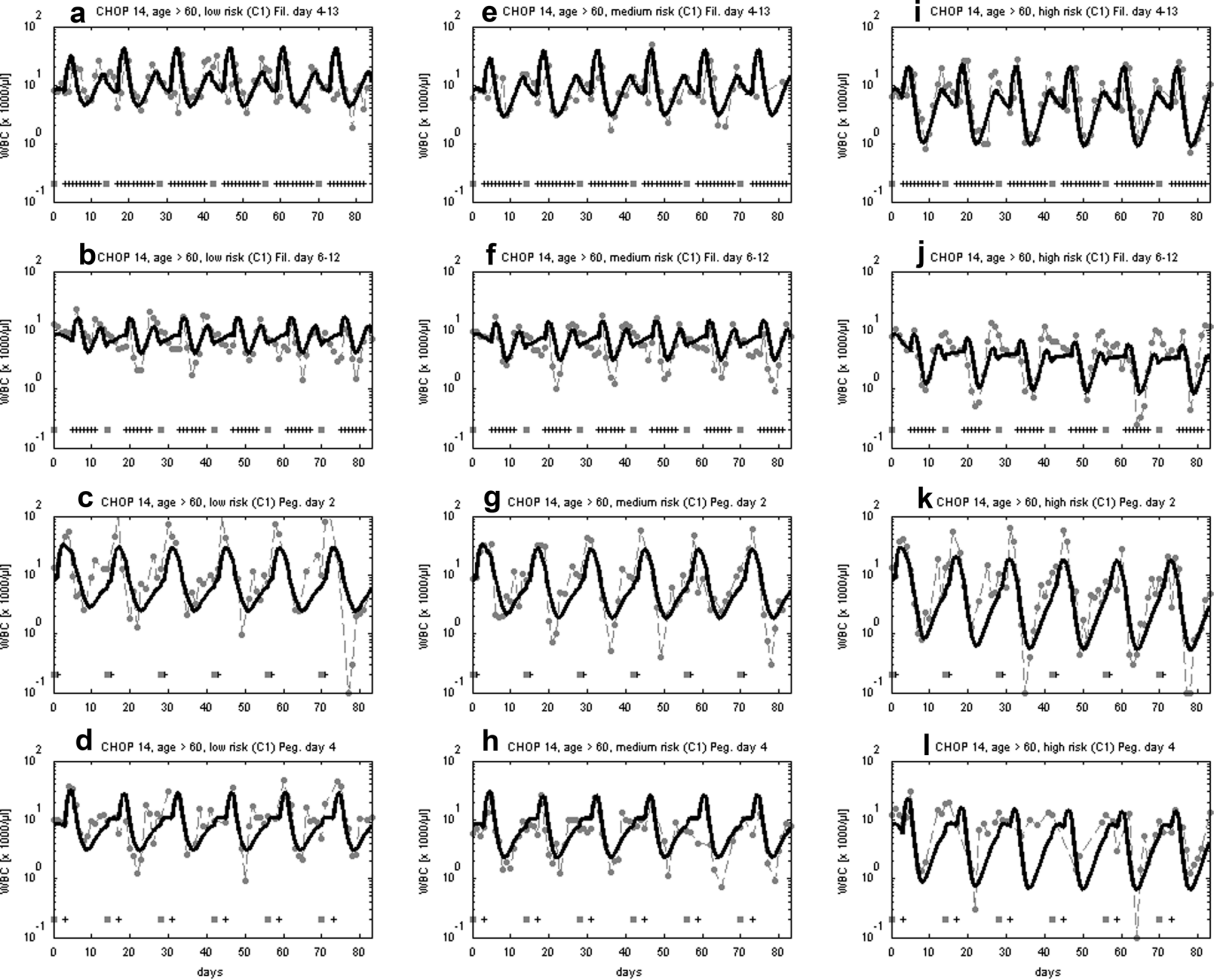
Agreement of model and data for CHOP-14 considering three risk groups and four G-CSF schedules. We consider six cycles of CHOP-14 chemotherapy of non-Hodgkin’s lymphoma for elderly patients (age > 60) at low leukopenic risk (**a**–**d**), medium leukopenic risk (**e**–**h**), and high leukopenic risk (**i**–**l**). Data and model prediction are compared for filgrastim on days 4–13 or 6–12 and pegfilgrastim on day 2 or 4. Dots represent patient medians, squares correspond to chemotherapy administrations, “+” corresponds to days with G-CSF injections

Assuming that interindividual heterogeneity in toxic response can be traced back to differences in chemotherapy toxicity parameters rather than cell-kinetic parameters, we estimated these parameters for the three risk groups considered. Resulting agreement of model and data can be found in Fig. 6.

**Fig. 7:**
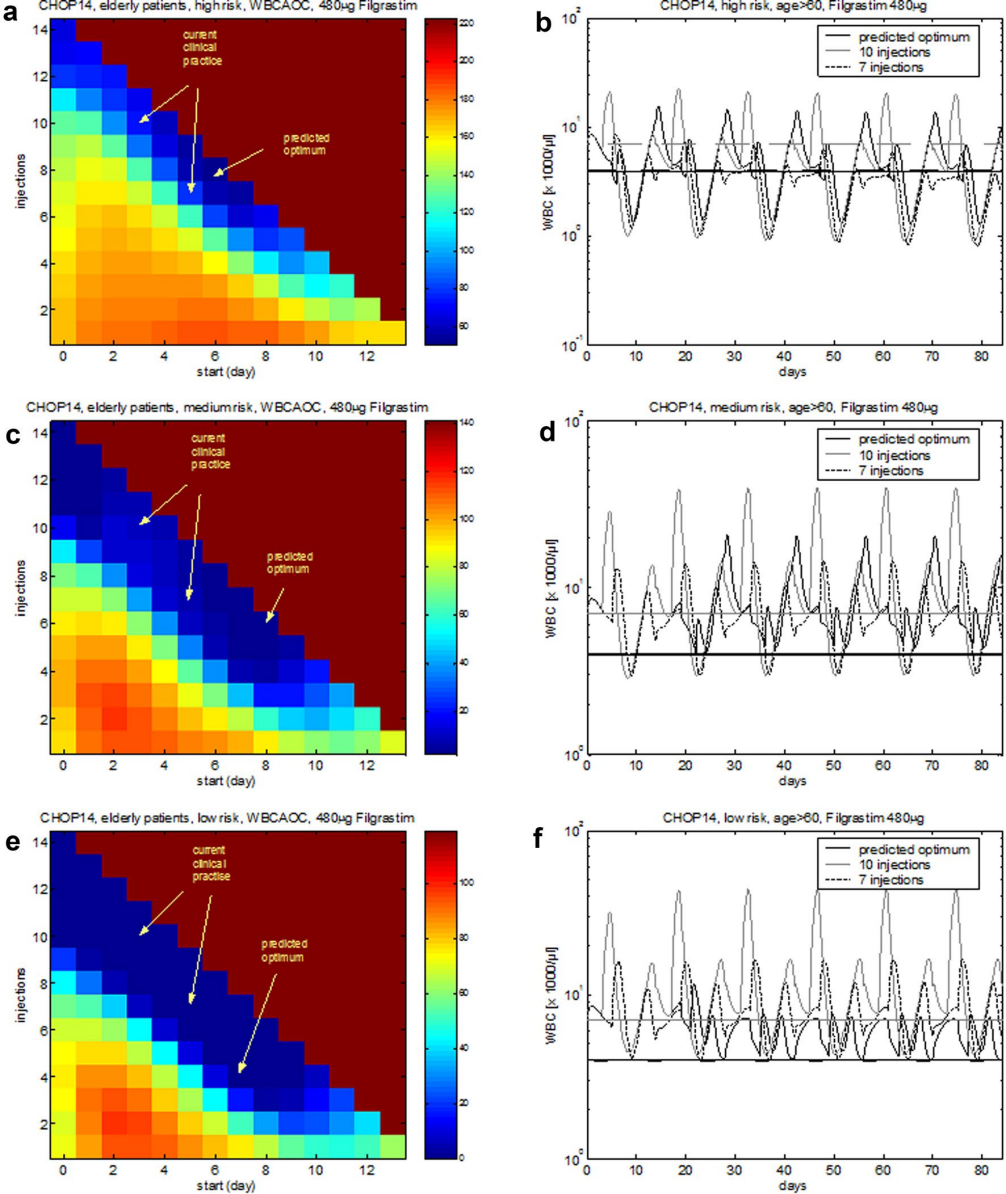
Risk-specific G-CSF schedules for CHOP-14 chemotherapy of elderly patients. Optimization is performed for cycles 2–6 since first cycle toxicity is included into the risk model. **a**, **b** High-risk, **c**, **d** medium-risk, **e**, **f** low-risk group. **a**, **c**, **e** The colour corresponds to the predicted WBCAOC (blue: lower WBCAOC—lower toxicity, red: high WBCAOC—higher toxicity). The *X*-axis corresponds to the starting day of filgrastim treatment. The *Y*-axis represents the number of filgrastim injections. Background colour: toxicity obtained without G-CSF treatment. Panels **b**, **d** and **f** show the WBC time course of the predicted optimal schedule in comparison to the current standard

Estimated toxicity parameters can be used to make risk-specific predictions regarding specific optimal G-CSF schedules. We conclude that for the highest risk group, filgrastim treatment should be started around d6–8 after start of therapy cycle with at least eight injections (Fig. 7a, b). For the medium- and low-risk groups, timing is less important and 6, respectively, 4 injections result in sufficient recovery (Fig. 7c–f).

Pegfilgrastim should be injected around d6–7 after chemotherapy in the high-risk and medium-risk group. For the low-risk group, timing is not important (Fig. S2b, d, f in Additional file 1). Regarding pegfilgrastim dosing, we predict that the low-risk group can safely be treated with considerably less pegfilgrastim (Fig. S2e in Additional file 1). To a lesser extent, this also applies for the medium- and high-risk group if pegfilgrastim is administered within the above-mentioned optimal time interval (Fig. S2a, c in Additional file 1).

## Discussion

Although the haematopoietic growth factor G-CSF is routinely applied in clinical practice since many years, its optimal use in a given clinical situation is often unknown or not well investigated. The reason is that the performance of alternative G-CSF schedules is difficult to predict in view of the strong interaction of chemotherapy-induced leukopenia, pharmacokinetic properties of G-CSF and the resulting effects on bone marrow leukopoiesis.

In view of the large number of variable therapy options (dosing and scheduling of cytotoxic drugs and G-CSF, different G-CSF pharmaceuticals, individual risk factors of patients), it is practically impossible to study this problem solely on the basis of clinical trials. Thus, there is a strong need for predictive modelling of G-CSF applications. Pastor et al. (2015) proposed a statistical model, while Quartino et al. (2014) proposed a semi-mechanistic model for this purpose. Craig et al. (2015) used their granulopoiesis model to explore alternative filgrastim schedules for general 14-day chemotherapy cycles. Here we propose to use our recently established biomathematical model of human granulopoiesis under G-CSF and chemotherapy treatments to address this task. Our model is based on biological assumptions on bone marrow haematopoiesis, PK and PD effects of G-CSF injections and the cytotoxic effects of chemotherapy.

The model was developed on the basis of large clinical and literature data sets (Schirm et al. 2013, 2014a, b). To apply the model, it is necessary to estimate the bone marrow toxicity of an applied cytotoxic drug or drug combination, which can be achieved by studying time-series data of patients treated under this condition. By this approach, we were able to quantify bone marrow toxicities of a total of 10 drugs and 33 schedules (Schirm et al. 2014b). After quantifying the toxicity, the performance of alternative G-CSF schedules can be simulated by the model. We already applied this method in the planning phase of a number of clinical trials. Data collected under the newly proposed schedules showed that our predictions are in good agreement with the data. In view of these encouraging results, we propose additional optimized G-CSF schedules here. The proposed schedules are intended to be verified in clinical trials.

Different G-CSF derivatives are in practical use. Here we focused on filgrastim and pegfilgrastim which are generally considered as equally potent to prevent leukopenia if properly applied. A few studies and meta-analyses indicate advantages for pegfilgrastim (Clark et al. 2003, 2005; Cooper et al. 2011; Mhaskar et al. 2014; Lambertini et al. 2015). Indeed, pegfilgrastim can be applied more easily increasing compliance. But filgrastim can be dosed more precisely allowing individual adaptations. This is especially relevant for risk-adapted G-CSF treatments. Moreover, it is supposed that the amount of pegfilgrastim injected by a single standard syringe might be too high for some patients (Ishiguro et al. 2008; Djulbegovic et al. 2013; Masuda et al. 2015). Therefore, going beyond pure variation of starting time of pegfilgrastim, we also considered scenarios with reduced dosage of pegfilgrastim.

We studied different outcomes to assess the resulting cytotoxic outcome of a schedule, namely WBCAOC, DoL and MLC. Pros and cons of these outcomes are discussed elsewhere (Scholz et al. 2006) and we propose WBCAOC as the most reasonable choice. This allows us to compare different G-CSF schedules with respect to their expected cytotoxic outcome, and finally, to optimize the schedules. The relationship between the degree of chemotherapy-induced leukopenia and resulting risk for infections is well-established (Colotta et al. 1992; Bennett et al. 2013; Li et al. 2016).

As practical applications of our model, we considered for example different starting times of pegfilgrastim for the adjuvant breast cancer chemotherapy ETC in the patient population studied in Moebus et al. (2010). According to our simulations, we predict that the application at d4 after chemotherapy is superior to d2 and that d6 is optimal. However, the differences are small. Moreover, the strongest leukopenic risk is expected for the cycles with cyclophosphamide applications. Here, the nadir occurs in a narrow time interval which might be difficult to capture in a clinical trial. This could explain the results of Loibl et al. (2011) who observed a (non-significant) trend towards better performance of the d4 schedule compared to d2.

For the BEACOPP escalated regimen to treat advance stage Hodgkin’s lymphoma (Diehl et al. 2003), we predict that pegfilgrastim is optimally applied at d6–7 after chemotherapy. However, this would still fall into the period of procarbacine treatment.

We also propose optimized filgrastim treatment for three scenarios: for BEACOPP escalated, we predict that starting 1 day earlier and increasing the number of G-CSF injections would result in improved leukopenia prophylaxis. For the ETC chemotherapy mentioned above, we predict that filgrastim d7–14 after chemotherapy is clearly superior to the current standard d4–11. But since the haematotoxic risk clearly depends on the applied drugs, we also considered different filgrastim schedules for cycles 1–3 (epirubicin), 4–6 (paclitaxel) and 7–9 (cyclophosphamide), respectively. However, only small improvements were predicted compared to the d7–14 schedule.

Since time-intensified CHOP is advantageous for the treatment of high-grade non-Hodgkin’s lymphoma in elderly patients (Pfreundschuh et al. 2004a; Roesch et al. 2014; Rosch et al. 2016), we designed a regimen with six cycles of CHOP repeated every 12 days (CHOP-12). We predict that with optimal filgrastim support at d7–12, the toxicity might be tolerable but slightly cumulates over six cycles.

Another application of the model is the development of risk-adapted G-CSF schedules as recommended (Kuderer et al. 2006; Georgala and Klastersky 2015). This is achieved under the assumption that risk groups differ in sensitivity to chemotherapeutic drugs rather than response to G-CSF treatment (Chatta et al. 1994). We established a statistical model of leukopenia risk, depending on pre-therapeutical (i.e. age, sex) and intra-therapeutical (observed toxicity in first cycle) risk factors for patients of high-grade non-Hodgkin’s lymphoma in the past (Ziepert et al. 2008). However, the risk score did not result in recommendations regarding individualized G-CSF regimen so far. We addressed this issue in our paper by dividing patients into tertiles for which we propose specific G-CSF schedules. Indeed, we could detect some potential for risk-dependent filgrastim treatment: For the optimal schedules, number of filgrastim injections differed between four for the low-risk group, six for the medium-risk group and eight for the high-risk group. No optimization potential was detected for single pegfilgrastim injections (optimum d6–7 after chemotherapy for all risk groups). This approach can be generalized to other therapy schedules for which a leukopenia risk score is available.

As a general recommendation observed throughout our scenarios, we conclude that filgrastim and pegfilgrastim treatment should not be started too early after chemotherapy. The major reason is that G-CSF releases the bone marrow reserve of granulocytes which should be avoided if the number of granulocytes is still sufficiently high. However, this might be applicable only for intense chemotherapies with a high risk of leukopenia (Whitworth et al. 2009; Cheng et al. 2014). Moreover, filgrastim should not be stopped too early. Even if granulocytes are recovered, we predict a benefit of maintained G-CSF treatment in the subsequent chemotherapy cycle. According to our model simulations, we also expect that there is some potential to reduce the dose of single pegfilgrastim injections without loss of efficacy. However, this prediction must be considered with caution since it is based on extrapolation of absorption kinetics.

A limitation of our method is that we only consider the number of leukocytes and not the clinically more relevant outcome of infection. Although there are strong relationships between leukocyte counts and risk for infection (Bennett et al. 2013; Colotta et al. 1992), our method does not account for leukocyte function or other measures to prevent infections such as prophylactic antibiotic treatment or hospitalization. Another limitation is that we optimized G-CSF therapy for the medians of patient populations or risk groups while patient extremes are most relevant. We aim at addressing this issue by modelling individual time courses in the future.

## Conclusions

We conclude that we established a biomathematical model of human granulopoiesis under chemotherapy which allows predictions of yet untested G-CSF schedules, comparisons between them, and with it, optimization of filgrastim and pegfilgrastim treatment. Some model predictions were already validated in clinical trials. We provided a number of additional suggestions for optimized G-CSF schedules for chemotherapies of different diseases and risk groups. As a general rule of thumb, G-CSF treatment should not be started too early and patients could profit from filgrastim treatment continued until the end of the chemotherapy cycle.

## Electronic supplementary material

Below is the link to the electronic supplementary material.

Additional file 1: Modelling chemotherapy effects on granulopoiesis: Supplement Material. The file Additional file 1 contains model parameters and further optimization results. (PDF 460 kb)
