# Supplementary material for: Model-based optimization of G-CSF treatment during cytotoxic chemotherapy
Source: J Cancer Res Clin Oncol. 2017 Nov 4;144(2):343–58. doi: 10.1007/s00432-017-2540-1 (PMC5794835; doi:10.1007/s00432-017-2540-1)
Supplement: Supplementary file 1 — Additional file 1: Modelling chemotherapy effects on granulopoiesis: Supplement Material. The file Additional file 1 contains model parameters and further optimization results. (PDF 460 kb) [file 432_2017_2540_MOESM1_ESM.pdf]

Optimizing G-CSF schedules during chemotherapy: supplement material

Sibylle Schirm,[1] Christoph Engel,[1] Sibylle Loibl,[2] Markus Loeffler,[1] and Markus Scholz[1*]

[1] Institute for Medical Informatics, Statistics and Epidemiology (IMISE), Medical Faculty, University of Leipzig, Germany, Haertelstraße 16-18, 04107 Leipzig

[2] German Breast Group, c/o GBG Forschungs GmbH, Martin-Behaim-Straße 12, 63263, Neu-Isenburg, Germany

* Corresponding author: Markus Scholz, Institute for Medical Informatics, Statistics and Epidemiology (IMISE), Medical Faculty, University of Leipzig, Germany, Haertelstraße 16-18, 04107 Leipzig, e-mail: mscholz@imise.uni-leipzig.de

Table S1. Data sets. We present data sets used for model development and validation

Disease	Cyctotoxic drugs modelled	Chemotherapy schemes	G-CSF	Reference
Breast cancer	Doxorubicin, Docetaxel, Epirubicin, Paclitaxel, Cyclophosphamid	TA, EC-T , E-T-C	Filgrastim, Pegfilgrastim	(Moebus et al. 2010; Holmes et al. 2002; Yowell and Blackwell 2002; Zamboni 2003)
NSCLC	Carboplatin, Paclitaxel	CP	Filgrastim, Pegfilgrastim	
DLBCL	Cyclophosphamid, Doxorubicin, Prednison, Vincristine	R CHOP-14	Pegfilgrastim	(Brusamolino et al. 2006; Mey et al. 2007)
NHL	Cyclophosphamid, Doxorubicin, Etoposide, Prednison, Vincristine	high-CHOEP-14, high-CHOEP-21, CHOP-14, CHOEP-14, CHOP-21, CHOEP-21, R-CHOP-14	Filgrastim	(George et al. 2003; Pfreundschuh et al. 2008; Pfreundschuh et al. 2004a; Pfreundschuh et al. 2004b; Trumper et al. 2008; Zwick et al. 2011)
HD	Cyclophosphamid, Doxorubicin, Etoposide, Prednison, Procarbacine, Bleomycin, Vincristine	BEACOPP-14, BEACOPP-21, BEACOPP-21 escalated	Filgrastim	(Diehl et al. 2003; Sieber et al. 2003)
relapsed or persistent HD or NHL	Etoposide, Cisplatin, Cytarabin, Methyl-prednisolon	ESHAP	Filgrastim, Pegfilgrastim	

Table S2. Toxicity parameters. We present estimated toxicity parameters on stem cells (S), progenitors (CG), proliferating precursors (PGB), maturing precursors (MGB) and lymphocytes (LYM). Toxicity parameters are specific for cytotoxic drug concentrations and risk groups. CHOP chemotherapy consists of Cyclophosphamid (750 mg/m²), Doxorubicin (50 mg/m²), and Vincristine (2 mg).

CHOP chemotherapy	S	CG	PGB	MGB	LYM
high risk	0.2161	1.1848	0.6751	0.0002	20.0000
medium risk	0.2161	0.3697	0.2343	0.0002	12.6982
low risk	0.2152	0.1301	0.1197	0.0001	9.8753

Table S3. Outcome values of different simulated G-CSF schedules, chemotherapies and risk groups.

Therapy	risk group	Outcome measure	Optimal start of Fil	Optimal #Injection Fil	Optimal outcome value Fil	Optimal start of Peg	Optimal outcome value Peg	Currently used schedules	Current Outcome Value
CHOP-14 elderly	high	WBCAOC	7	8	50.51	7	57.16	Fil d4-13	72.58
								Fil d6-12	77.92
								Peg d2	112.45
								Peg d4	91.10
		DoL	6	9	27.17	6	29.88	Fil d4-13	34.67
								Fil d6-12	56.17
								Peg d2	47.67
								Peg d4	39.50
		MLC	9	5	0.99	7	1.15	Fil d4-13	0.88
								Fil d6-12	0.82
								Peg d2	0.56
								Peg d4	0.70
	medium	WBCAOC	9	6	2.16	7	2.81	Fil d4-13	10.47
								Fil d6-12	7.55
								Peg d2	38.82
								Peg d4	22.14
		DoL	9	6	2.75	7	7.13	Fil d4-13	15.00
								Fil d6-12	11.50
								Peg d2	28.79
								Peg d4	21.29
		MLC	8	4	2.84	7	3.21	Fil d4-13	2.84
								Fil d6-12	2.84
								Peg d2	1.90
								Peg d4	2.46
	low	WBCAOC	8	4	0.00	7	0.00	Fil d4-13	0.00
								Fil d6-12	0.00
								Peg d2	21.06
								Peg d4	6.94
		DoL	8	4	0.00	7	0.00	Fil d4-13	0.00
								Fil d6-12	0.00
								Peg d2	22.29
								Peg d4	14.29
		MLC	8	5	4.45	7	4.38	Fil d4-13	4.25
								Fil d6-12	4.01

								Peg d2	2.51
								Peg d4	3.21
BEACOPP esc	all	WBCAOC	7	15	62.07	7	52.98	Fil d8-15	145.16
		DoL	7	15	32.17	6	28.17	Fil d8-15	76.04
		MLC	7	13	0.67	7	0.56	Fil d8-15	0.35
ETC	all	WBCAOC	7	8	0.70	6	5.34	Fil d3-10	14.86
		DoL	7	8	3.63	6	5.54	Fil d3-10	11.71
		MLC	7	8	3.53	7	2.51	Fil d3-10	1.80
CHOP-12	all	WBCAOC	7	6	19.12	6	20.50	-	-
		DoL	6	7	17.5	6	15.54	-	-
		MLC	7	6	2.05	6	1.93	-	-

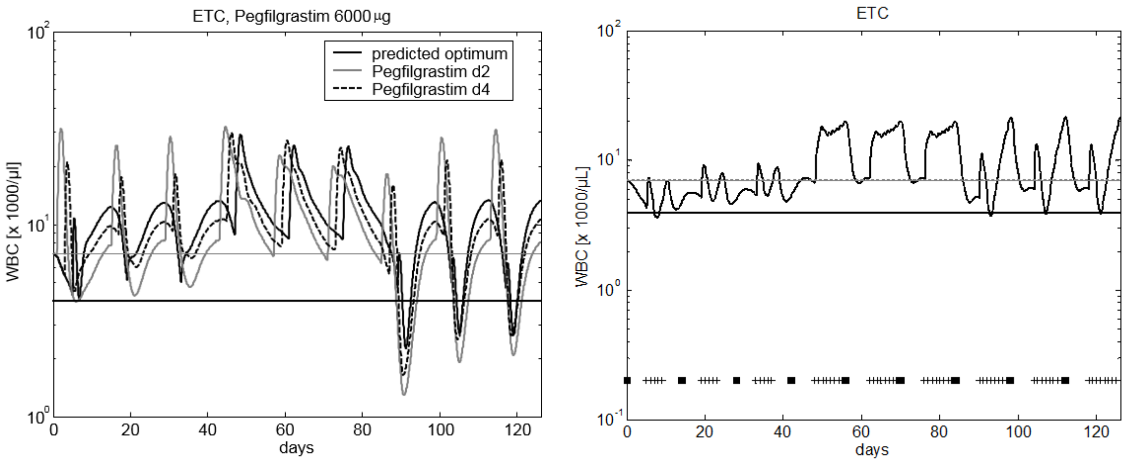

Figure S1. Model predictions of alternative G-CSF schedules for the adjuvant breast cancer therapy ETC. (Left) Pegfilgrastim 6mg d2, d4 and d6 (optimal). (Right) cycle-wise optimization of Filgrastim starting on day 6 with five injections at cycles 1-3 and on day 7 with eight injections at cycles 4-9.

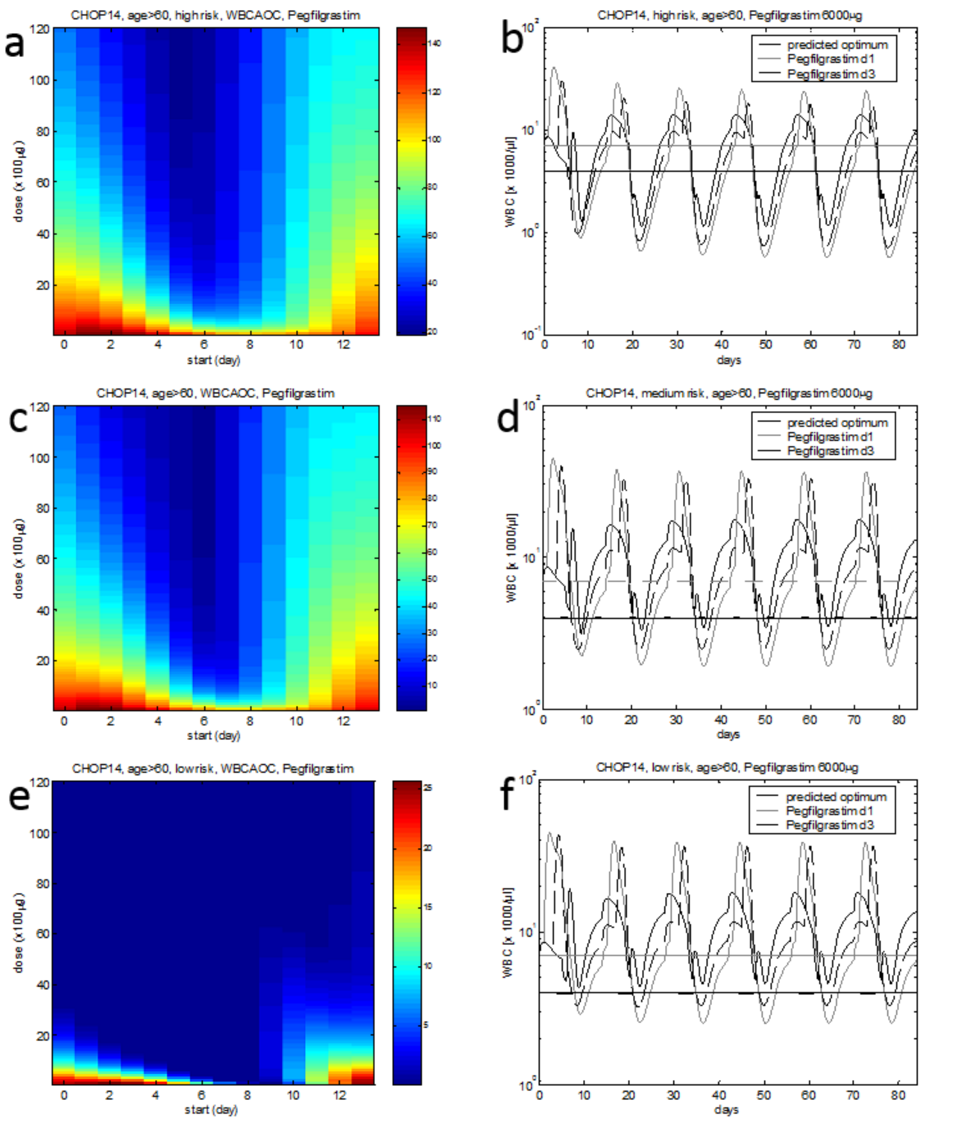

Figure S2. Prediction of leukopenic outcome of modified Pegfilgrastim schedules, dependent on risk group. We analysed 6 cycles of CHOP-14, elderly patients (age>60) for the three risk groups considered. Starting time and dosage of single Pegfilgrastim injections per cycle are varied. Panels on the left present WBCAOC of leukocytes as primary outcome. Panels on the right present the predicted time courses of leukocytes for the optimal timing and the timing day 1 and 3 of 6000µg Pegfilgrastim (a-b) high risk group, (c-d) medium risk group, (e-f) low risk group.

References

Brusamolino E, Rusconi C, Montalbetti L, Gargantini L, Uziel L, Pinotti G, Fava S, Rigacci L, Pagnucco G, Pascutto C, Morra E, Lazzarino M (2006) Dose-dense R-CHOP-14 supported by pegfilgrastim in patients with diffuse large B-cell lymphoma: a phase II study of feasibility and toxicity. Haematologica 91(4):496–502

Diehl V, Franklin J, Pfreundschuh M, Lathan B, Paulus U, Hasenclever D, Tesch H, Herrmann R, Dorken B, Muller-Hermelink H, Duhmke E, Loeffler M, German Hodgkin's Lymphoma Study Gr, [Nachname nicht vorhanden] (2003) Standard and increased-dose BEACOPP chemotherapy compared with COPP-ABVD for advanced Hodgkin's disease. New Engl J Med 348(24):2386–2395. doi: 10.1056/Nejmoa022473

George S, Yunus F, Case D, Yang BB, Hackett J, Shogan JE, Meza LA, Neumann TA, Liang BC (2003) Fixed-dose pegfilgrastim is safe and allows neutrophil recovery in patients with non-Hodgkin's lymphoma. Leukemia & lymphoma 44(10):1691–1696. doi: 10.1080/1042819031000063462

Holmes FA, Jones SE, O'Shaughnessy J, Vukelja S, George T, Savin M, Richards D, Glaspy J, Meza L, Cohen G, Dhami M, Budman DR, Hackett J, Brassard M, Yang BB, Liang BC (2002) Comparable efficacy and safety profiles of once-per-cycle pegfilgrastim and daily injection filgrastim in chemotherapy-induced neutropenia: a multicenter dose-finding study in women with breast cancer. Annals of oncology : official journal of the European Society for Medical Oncology / ESMO 13(6):903–909

Mey UJ, Maier A, Schmidt-Wolf IG, Ziske C, Forstbauer H, Banat GA, Reber M, Strehl JW, Gorschlueter M (2007) Pegfilgrastim as hematopoietic support for dose-dense chemoimmunotherapy with R-CHOP-14 as first-line therapy in elderly patients with diffuse large B cell lymphoma. Supportive care in cancer : official journal of the Multinational Association of Supportive Care in Cancer 15(7):877–884. doi: 10.1007/s00520-006-0201-z

Moebus V, Jackisch C, Lueck H, Du Bois A, Thomssen C, Kurbacher C, Kuhn W, Nitz U, Schneeweiss A, Huober J, Harbeck N, Minckwitz G von, Runnebaum IB, Hinke A, Kreienberg R, Konecny GE, Untch M (2010) Intense dose-dense sequential chemotherapy with epirubicin, paclitaxel, and cyclophosphamide compared with conventionally scheduled chemotherapy in high-risk primary breast cancer: mature results of an AGO phase III study. J Clin Oncol 28(17):2874–2880. doi: 10.1200/JCO.2009.24.7643

Pfreundschuh M, Trumper L, Kloess M, Schmits R, Feller AC, Rube C, Rudolph C, Reiser M, Hossfeld DK, Eimermacher H, Hasenclever D, Schmitz N, Loeffler M (2004a) Two-weekly or 3-weekly CHOP chemotherapy with or without etoposide for the treatment of elderly patients with aggressive lymphomas: results of the NHL-B2 trial of the DSHNHL. Blood 104(3):634–641. doi: 10.1182/blood-2003-06-2095

Pfreundschuh M, Trumper L, Kloess M, Schmits R, Feller AC, Rudolph C, Reiser M, Hossfeld DK, Metzner B, Hasenclever D, Schmitz N, Glass B, Rube C, Loeffler M (2004b) Two-weekly or 3-weekly CHOP chemotherapy with or without etoposide for the treatment of young patients with good-prognosis (normal LDH) aggressive lymphomas: results of the NHL-B1 trial of the DSHNHL. Blood 104(3):626–633. doi: 10.1182/blood-2003-06-2094

Pfreundschuh M, Schubert J, Ziepert M, Schmits R, Mohren M, Lengfelder E, Reiser M, Nickenig C, Clemens M, Peter N, Bokemeyer C, Eimermacher H, Ho A, Hoffmann M, Mertelsmann R, Trumper L, Balleisen L, Liersch R, Metzner B, Hartmann F, Glass B, Poeschel V, Schmitz N, Ruebe C, Feller AC, Loeffler M, German High-Grade Non-Hodgkin Lymphoma Study, Group (2008) Six versus eight cycles of bi-weekly CHOP-14 with or without rituximab in elderly patients with aggressive CD20+ B-cell lymphomas: a randomised controlled trial (RICOVER-60). The lancet oncology 9(2):105–116. doi: 10.1016/S1470-2045(08)70002-0

Sieber M, Bredenfeld H, Josting A, Reineke T, Rueffer U, Koch T, Naumann R, Boissevain F, Koch P, Worst P, Soekler M, Eich H, Muller-Hermelink HK, Franklin J, Paulus U, Wolf J, Engert A, Diehl V (2003) 14-day variant of the bleomycin, etoposide, doxorubicin, cyclophosphamide, vincristine, procarbazine, and prednisone regimen in advanced-stage Hodgkin's lymphoma: Results of a pilot study of the German Hodgkin's Lymphoma Study Group. J Clin Oncol 21(9):1734–1739. doi: 10.1200/Jco.2003.06.028

Trumper L, Zwick C, Ziepert M, Hohloch K, Schmits R, Mohren M, Liersch R, Bentz M, Graeven U, Wruck U, Hoffmann M, Metzner B, Hasenclever D, Loeffler M, Pfreundschuh M, German High-Grade Non-Hodgkin's Lymphoma Study, Group (2008) Dose-escalated CHOEP for the treatment of young patients with aggressive non-Hodgkin's lymphoma: I. A randomized dose escalation and feasibility study with bi- and tri-weekly regimens. Annals of oncology : official journal of the European Society for Medical Oncology / ESMO 19(3):538–544. doi: 10.1093/annonc/mdm497

Yowell SL, Blackwell S (2002) Novel effects with polyethylene glycol modified pharmaceuticals. Cancer Treat Rev 28:3–6

Zamboni WC (2003) Pharmacokinetics of pegfilgrastim. Pharmacotherapy 23(8 Pt 2):9S–14

Zwick C, Hartmann F, Zeynalova S, Poschel V, Nickenig C, Reiser M, Lengfelder E, Peter N, Schlimok G, Schubert J, Schmitz N, Loeffler M, Pfreundschuh M, German High-Grade Non-Hodgkin Lymphoma Study, Group (2011) Randomized comparison of pegfilgrastim day 4 versus day 2 for the prevention of chemotherapy-induced leukocytopenia. Annals of oncology : official journal of the European Society for Medical Oncology / ESMO 22(8):1872–1877. doi: 10.1093/annonc/mdq674
